# A study on the proliferation of *Myzus persicae* (sulzer) during the winter season for year-round production within a smart farm facility

**DOI:** 10.1371/journal.pone.0276520

**Published:** 2022-10-21

**Authors:** Jae-Hoon Park, Jung-Min Lee, Eui-Joo Kim, Ji-Won Park, Eung-Pill Lee, Soo-In Lee, Young-Han You

**Affiliations:** 1 Department of Life Science, Kongju National University, Gongju, South Korea; 2 National Ecosystem Survey Team, National Institute of Ecology, Seochon, South Korea; 3 Invasive Alien Species Research Team, National Institute of Ecology, Seochon, South Korea; Zhejiang University, CHINA

## Abstract

In this study, we examined the feasibility of *Myzus persicae* proliferation through interrelationships with host plants in a smart farm facility during winter. We investigated aphid proliferation under an LED artificial light source and attempted to interpret aphid proliferation in relation to the net photosynthetic rate of the host plant, *Eutrema japonicum*. We observed that aphids continuously proliferated in the smart farm facility in winter without dormancy. The average number of aphids was greater under the 1:1 red:blue light irradiation time ratio, where the photosynthetic rate of the host plant was lower than under the 5:1 and 10:1 red:blue light irradiation time ratios. These results show that it is important to maintain a low net photosynthetic rate of the host plant, *E*. *japonicum*, in order to effectively proliferate aphids under artificial light such as in the case of smart farm facilities.

## Introduction

Crops are cultivated throughout the year in crop cultivation facilities, including in South Korea, owing to the increasing demand for vegetables due to economic development and improvement in national income [1, 2]. Recently, due to problems of the aging agricultural population, decrease in the agricultural population and lands, and frequent occurrence of disasters attributed to global warming, there has been a stagnation of agricultural productivity, and crop cultivation has changed from outdoor crop cultivation to facility crop cultivation [3].

However, with the cultivation of facility crops comes an associated pest problem. Crop damage caused by pests is gradually becoming more severe worldwide [4]. Major insect pests are *Myzus persicae* and *Aphis gossypii* (Hemiptera: Aphididae), *Tetranychus urticae* and *T*. *kanzawai* (Trombidiformes: Tetranychidae), *Polyphagotarsonemus latus* (Trombidiformes: Tarsonemidae), *Liriomyza trifolii* (Diptera: Agromyzidae), *Palpita indica* (Lepidoptera: Pyralidae), *Frankliniella intonsa* and *F*. *occidentalis* (Thysanoptera: Thripidae), and *Bemisia tabaci* (Hemiptera: Aleyrodidae) [5–10].

Pest control through pesticides is used in agriculture to improve crop productivity and quality [11]; however, due to the excessive use of pesticides, side effects, such as the extermination of non-targeted species (beneficial insects), the emergence of resistant pests, environmental pollution, and the conversion of potential insects into pests, are also greatly increasing [12–15]. Therefore, augmentative biocontrols, which use natural predators for environmentally friendly crop production, are being developed [16].

Previously reported biological control technologies for aphids include banker plant technology that uses plants infected with *Aphidius colemani* (a parasitoid wasp) and aphids [17]. This technology removes aphids by growing plants infected with *A*. *colemani*-infected aphid mummies and healthy adult aphids in pots and placing the pots in the crop cultivation area of the facility [17]. Recently, the Goyang Agricultural Technology Center succeeded in mass proliferation of *A*. *colemani* using barley banker plants in South Korea. However, this technology is not effective for small aphids because *A*. *colemani* is not parasitic on them. Therefore, to biologically control various pests, including aphids, general predators that feed on aphids, such as ladybugs, should be used.

The ladybug (*Harmonia axyridis* (Pallas)) is a representative biocontrol agent and a general predator of aphids [18]. Ladybugs in temperate regions of northern hemisphere usually finish wintering in late spring and lay their eggs in April-May after mating [19]. However, female ladybugs do not have a steady food source, and if they starve, the percentage of mature ovarioles decreases, which can negatively impact their reproduction [20]. To increase the effectiveness of ladybug biocontrols, it is necessary to provide adults with sufficient food sources, such as aphids, at the time of egg-laying. Therefore, research on the mass proliferation of aphids, which are a common food resource for major biocontrol agents, including ladybugs, in winter should be conducted. However, in the past, it was difficult to establish the mass proliferation of aphids during the winter season because aphids usually winter in their natural environment.

Unlike general crop production facilities, the recently developed smart farm is a type of cultivation facility that is enclosed from the external environment, is not affected by the weather, and can automatically control the optimal environmental conditions for crop cultivation [21]. When LEDs are used as artificial light, different short wavelengths of light can be combined to produce and supply the desired light wavelengths that fit the specific purposes of plant cultivation. Light sources can be installed close to the plants being cultivated because the amount of heat generated by the LEDs is small [21]. Therefore, compared to conventional cultivation facilities, smart farms can be more effectively used for optimizing the proliferation of aphids and their predators, such as ladybugs, along with host plant cultivation throughout the year, through examining and setting conditions for smooth aphid proliferation irrespective of weather.

Light is an important environmental factor that influences aphid-host plant interactions. Red and blue light, which are commonly used artificial light sources in smart farms, are more efficient for plant photosynthesis than other light wavelengths, and their efficiency depends on the ratio of red to blue light [22–25]. The LED light quality can be precisely modulated and controlled in a smart farm. Therefore, in smart farms, LED light is the best light source for studying aphid responses to light properties. Owing to these advantages, LED technology has also been used for research on various plants [26–30].

Aphids should be continuously supplied to biocontrol agents because they are a major source of food for augmentative biocontrol agents as crop pests. However, this is impossible in temperate regions due to the winter season. In our previous study investigating the relationship between aphids and their main predator in East Asia, ladybugs, we observed the proliferation of aphids during the summer within a smart farm facility [31]; however, aphid proliferation during winter has not been studied. Therefore, this present study investigated aphid proliferation during winter using *Eutrema japonicum* (Miq.) Koidz. as their host plant in a smart farm with controlled environment, and analyzed the cause of changes in aphid proliferation in relation to the host plant’s net photosynthetic rate.

## Materials and methods

### Plant cultivation and smart farm environment treatment

The host plant supplied as a food source for the aphids is *E*. *japonicum*, a perennial semi-shaded plant that has been observed to have aphids attached to and is suitable for cultivation in smart farm facilities with lower light than outdoor environments [32–34]. *E*. *japonicum* seedlings were cultivated from November 2021 to March 2022 in a smart farm by selecting similar sized plants with 3–5 aphids attached, and then transplanting one individual per pot. In the three chambers installed in the smart farm (Parus Co., Shanghai, China), pots with transplanted *E*. *japonicum* (12 pots per chamber) were placed, and the day length was set to 16 h. The smart farm we used has been owned the ecology lab in Kongju National University and was permitted for use in experiments.

**Fig 1:**
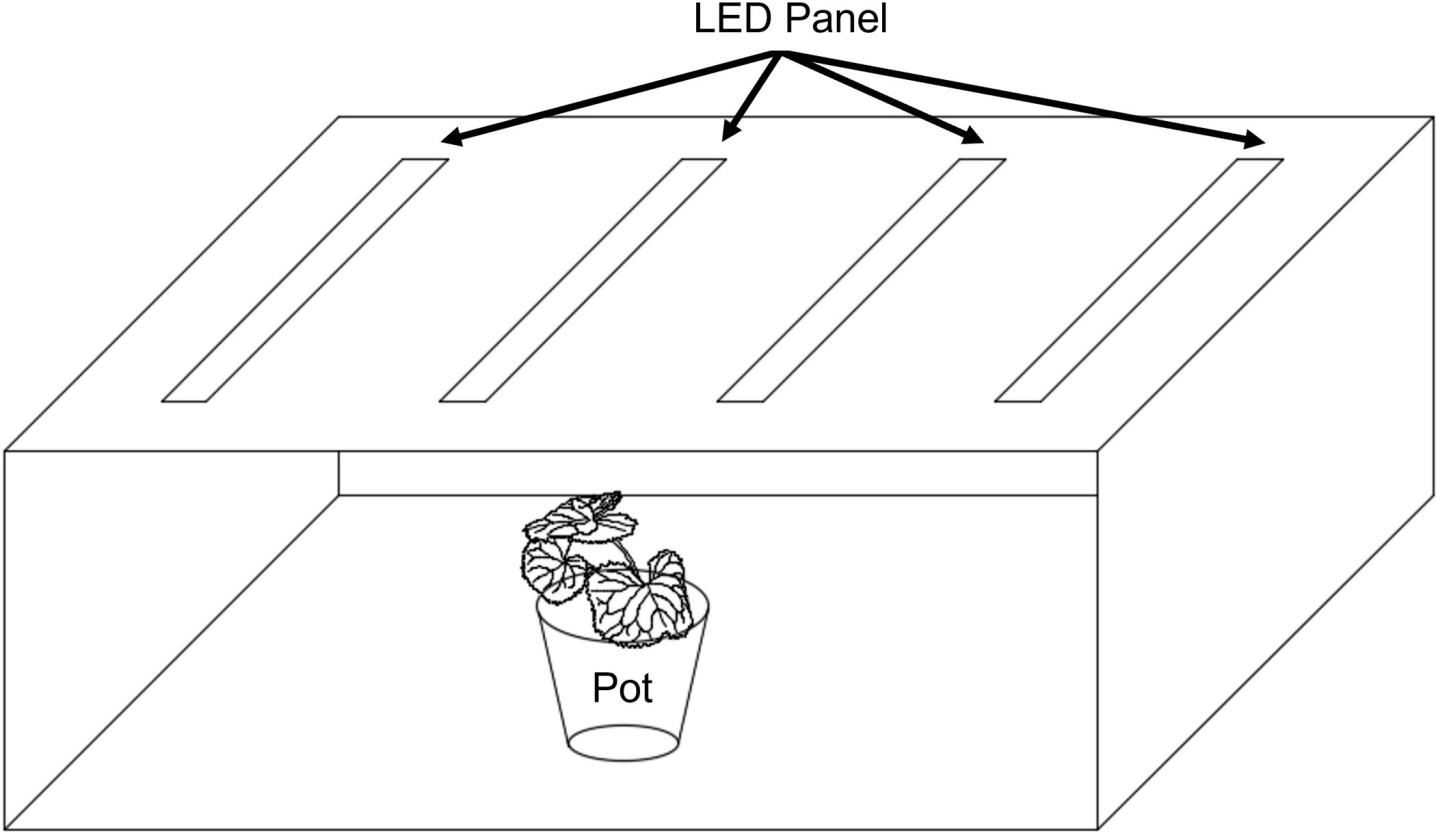
Schematic diagram of a light chamber (120cm × 52cm × 41.5cm) and a pot. The four LED lights, as the light sources, were set below the surface of the roof. The colors of all surfaces were white for light reflection.

The size of the smart farm was 360 cm (W) × 60 cm (L) × 230 cm (H). The size of each chamber positioned in the smart farm was 120 cm (W) × 52 cm (L) × 41.5 cm (H), and their walls were painted white to increase the light reflectance (Fig 1). In order to observe the aphid proliferation response according to light quality, different ratios of red (R, 660 nm) and blue (B, 450 nm) light efficient for plant photosynthesis was used for examining light quality inside the smart farm by controlling irradiation time of them [22, 35–40]. The irradiation time rates of R and B lights in the LED panels were set to 1:1, 5:1, and 10:1 (Table 1). In this case, the ratios of the amount of blue light / red light was 0.64 in RB(1:1), 0.13 in RB(5:1), 0.06 in RB(10:1), if calculated, because the number of LED units of red light was 33, and the number of LED units of blue light was 21 per chamber.

**Table 1:** Our previous studies that have investigated R:B ratio conditions in a smart farm facility.

Treatment^1^	Studied plant	Reference
**RB(1:1), RB(5:1), RB(10:1)**	*Eutrema japonicum*	[38]
	Six taxa of leafy vegetables	[39]
	*Lycium chinense*	[40]

^1^Represents 1:1, 5:1 and 10:1 of light irradiation time ratios of red:blue mixed light.

For *E*. *japonicum* cultivation, sand with the same particle size was placed in a circular pot of 15 cm (H) × 12 cm (D). The sand was mixed with bed soil (Heungnong bio, Monsanto Korea, Seoul, South Korea) at a ratio of 99.5:0.5 to supply the pot with nutrients. Water was supplied at intervals of 2–3 days so that the soil would not dry out, and the temperature and relative humidity were controlled using a thermo-hygrostat data logger LCSEMS (Parus Co., Shanghai, China). During the experimental period, light intensity in the smart farm was 101.44 ± 8.07 μmolm^-2^s^-1^, temperature was 11.15 ± 4.24°C, relative humidity was 50.84 ± 8.26%, and CO_2_ concentration was 355.18 ± 12.24 ppm (average ± standard deviation for all values).

### Measurement of aphid population

We cultivated *E*. *japonicum* with *M*. *persicae* placed in each chamber for one month to allow for acclimatization to the internal environment of the smart farm and then observed the number of aphids per leaf in each individual plant every month from December 2021 to March 2022. During this period, to determine whether there is a difference in the number of aphids depending on the location on the leaf, the number of aphids on the upper side (UPP) and lower side (UND) of the leaves was observed. Adult individuals of *Myzus persicae* were counted and divided into two groups, marking different external morphology: alate aphids (W) with a pair of wings, and apterous aphids (S) without wings.

### Measurement of the ecophysiological response of the host plant

To determine the change in the number of aphids in relation to the net photosynthetic rate (Pn; expressed in μmol m^-2^s^-1^) of the host plant, we measured the Pn of leaves to which aphids had been attached. Measurements were taken 39 times per chamber between 10 a.m. and 2 p.m. for a day in March 2022 using the photosynthesis measuring equipment LCi-SD Ultra Compact Photosynthesis System (ADC BioScientific Ltd., Hoddesdon, UK), which can measure photosynthetic factors, such as Pn.

To understand whether the leaf toughness of host plants affects the number of aphids on it, aphid-attached *E*. *japonicum* leaves with signs of feeding activity were sampled. Per chamber, nine leaf disks with a diameter of 5 mm were collected, and their dry weight was determined to calculate the leaf density, which is the leaf dry weight per constant area. It is known that increased leaf density increases leaf toughness [41], in turn affecting herbivorous pressure, e.g., by aphids, on the host plant.

### Statistical analysis

Statistical tests to compare among the light conditions for the Pn of *E*. *japonicum* and the number of leaves with *M*. *persicae* were performed using the one-way ANOVA test (p≤0.05), and the post hoc test was performed using the Fisher LSD method [42]. Factor analysis was performed to determine the relationship among the observed number of aphids on the upper side (UPP) and lower side (UND) of leaves, number of alate aphids (W) and apterous aphids (S), and net photosynthetic rate (Pn) of *E*. *japonicum* leaves attached with aphids [42]. When analyzing the factors, standardized data were used to eliminate errors caused by different units for each variable [42]. Statistical analyses were performed using Statistica (version 7) statistical package (StatSoft Inc., Tulsa, USA).

## Results

**Fig 2:**
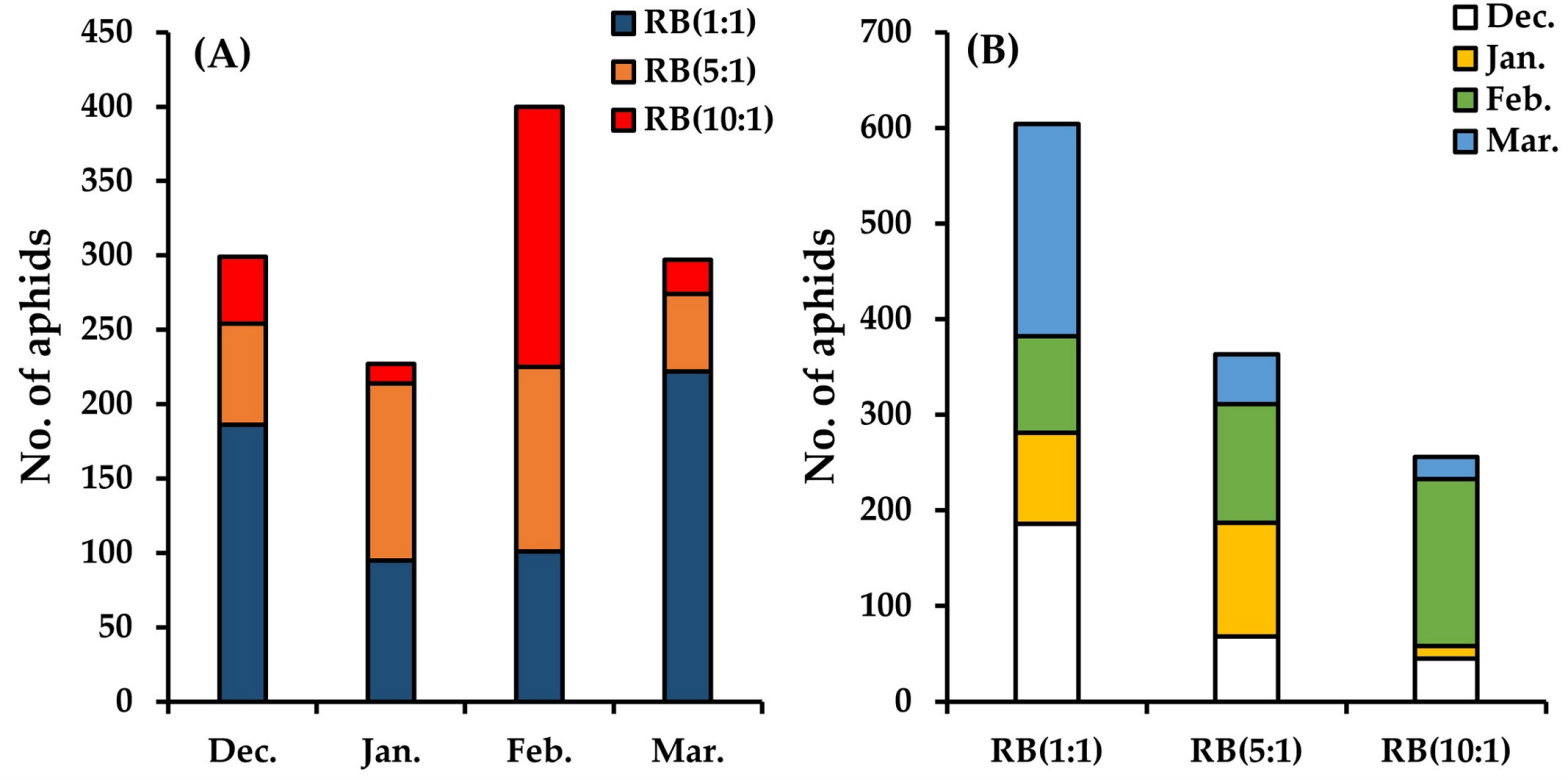
The number of aphids on the leaves of *Eutrema japonicum* monthly (A) and according to red:blue (RB) mixed light irradiation ratios of 1:1, 5:1 and 10:1 (B).

*M*. *persicae* was continuously observed from December 2021 to March 2022 under the light conditions of RB(1:1), RB(5:1), and RB(10:1) in the smart farm (S1 and S2 Tables), with highest frequency in February and lowest in January (Fig 2A). The number of aphids under light conditions was highest under RB(1:1) and lowest under RB(10:1) in December; in January, it was highest under RB(5:1) and lowest under RB(10:1); in February, it was highest under RB(10:1) and lowest under RB(1:1); and in March, it was highest under RB(1:1) and lowest under RB(10:1) (Fig 2).

**Fig 3:**
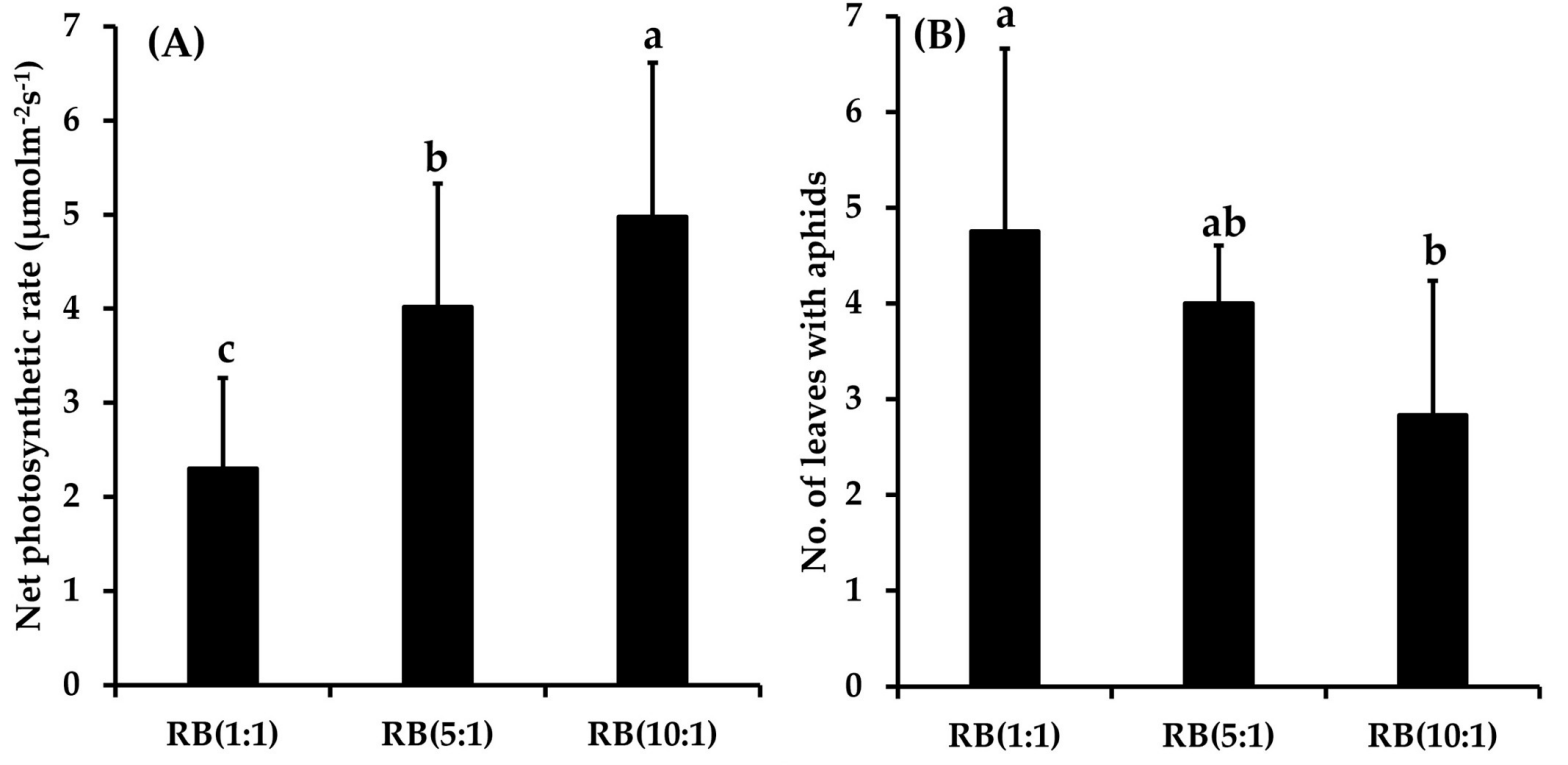
The net photosynthetic rates (A; μmolm^-2^s^-1^) and the number of leaves with aphids (B) during the study period of *Eutrema japonicum* under each light treatment condition. The error bars indicate standard deviation. RB represents ratio of red and blue mixed light. The lower case letters represent significant differences (p ≤ 0.05) among RB(1:1), RB(5:1), and RB(10:1).

**Fig 4:**
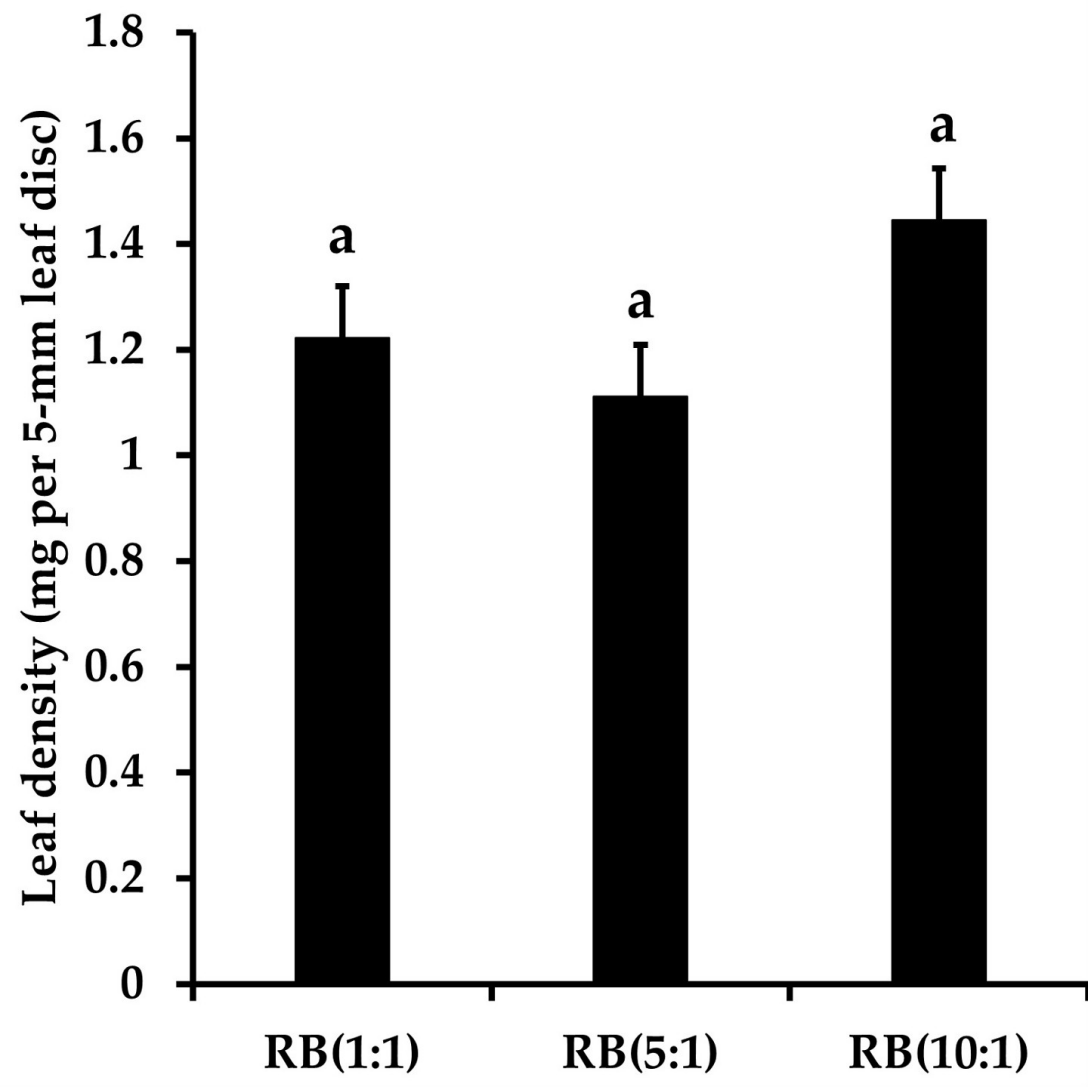
Leaf density (mg per 5-mm diameter leaf disc) measured by dry biomass of a leaf disc of *Eutrema japonicum* under each light treatment condition. RB indicates ratio of red and blue mixed light.

The Pn of *E*. *japonicum* was in the order RB(10:1) > RB(5:1) > RB(1:1) (Fig 3A and S3 and S4 Tables). This pattern was the opposite to that of the aphid population (Fig 2B). Additionally, the oscillation pattern of the number of leaves with aphids per individual *E*. *japonicum* plant matched that of aphid population size in the order RB(1:1) ≥ RB(5:1) ≥ RB(10:1) (Figs 2B and 3B and S5 Table). However, there was no difference in leaf density in relation to light quality (Fig 4).

**Fig 5:**
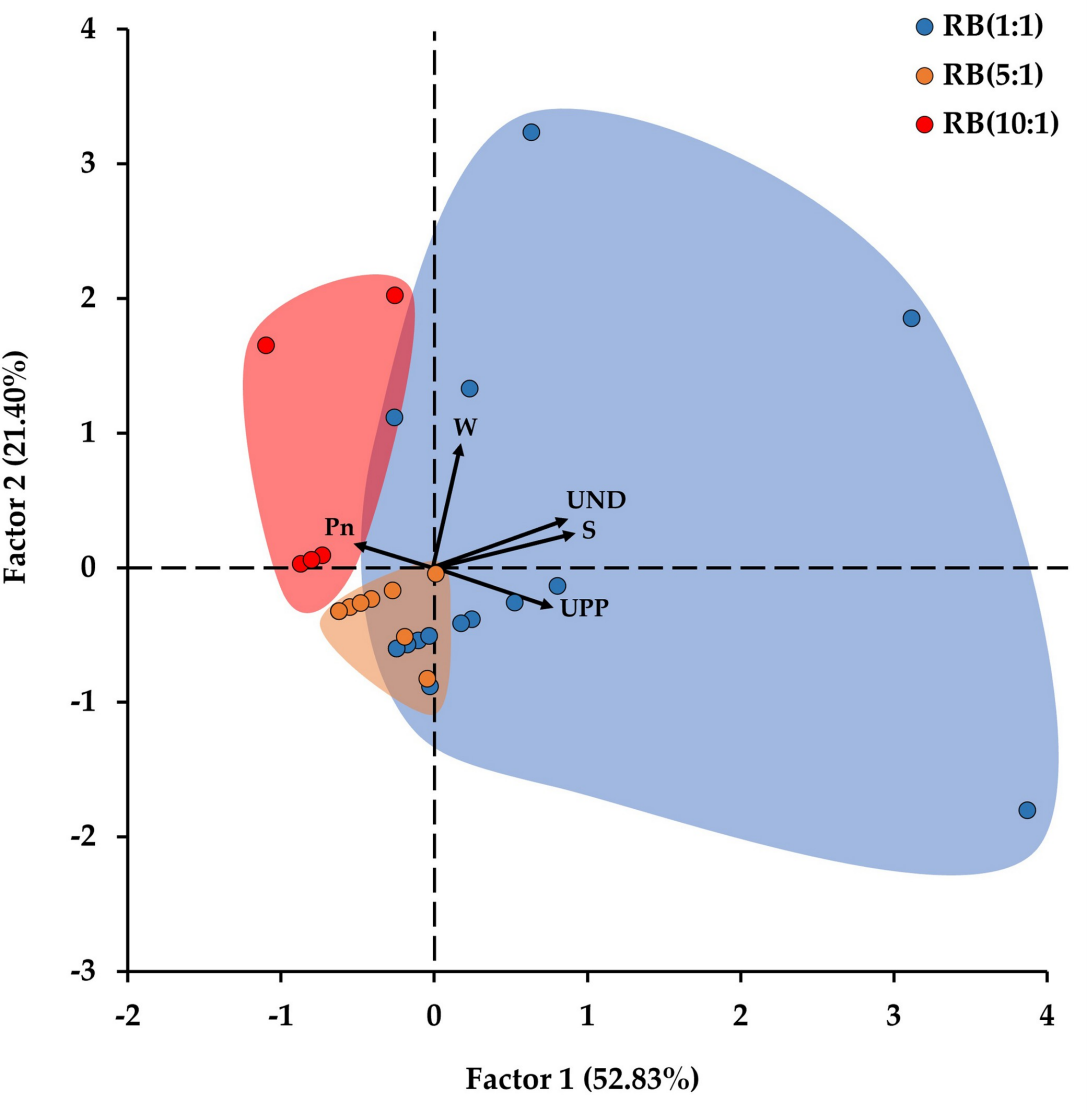
The factor score distribution of factor analysis, showing the relation between aphid and host plant, using net photosynthetic rate (Pn; μmolm^-2^s^-1^) of *Eutrema japonicum*, the number of alate (W) and apterous (S) aphids, and the number of aphids on the upper side (UPP) and lower side (UND) of *Eutrema japonicum* leaves. The arrows indicate the factor loadings on each variable. RB represents the ratio of red and blue mixed light.

Factor analysis using the number of aphids and the Pn of *E*. *japonicum* measured in March showed that the number of aphids was affected by light quality and Pn (Fig 5 and Table 2). When the distributed factor scores of RB(1:1), RB(5:1), and RB(10:1) were divided into three groups, their distribution positions were separated into factors 1 and 2 (Fig 5). Considering that a variable with factor loading of 0.3 or more is a major variable for factor 1 or 2 [43], the major variables affecting the distribution of the factor scores were UPP, UND, W, S, and Pn (Fig 5 and Table 2). Therefore, the characteristics of aphids were affected by the Pn of *E*. *japonicum*.

**Table 2:** The factor loadings determined for five measured variables of factor 1 and factor 2.

Variables	Abb.	Factor 1	Factor 2
**No. of aphids on the upper side of the leaves**	UPP	0.79	-0.29
**No. of aphids on the underside of the leaves**	UND	0.89	0.37
**No. of alate aphids**	W	0.19	0.93
**No. of apterous aphids**	S	0.94	0.25
**Net photosynthetic rate**	Pn	-0.50	0.18

## Discussion

*M*. *persicae* was continuously observed from December to March, which is the winter season in temperate Asia, under the light conditions of RB(1:1), RB(5:1), and RB(10:1) in the smart farm (Fig 2A). On average, the number of aphids per month increased by approximately 32-fold compared to the number of early aphids. However, in the wild environment within temperate Asia of northern hemisphere, aphids generally appear from late March or early April and have been observed to be active until late October or early November [44–46]. This result was inconsistent with the previously reported life cycle of *M*. *persicae* in the wild, as this species lays eggs in November and winters them until March-April of the following year [44]. Therefore, when *M*. *persicae* were supplied with *E*. *japonicum* as a host plant in a smart farm, we observed continuous proliferation over time irrespective the aphid life cycle in the wild environment.

In this regard, *M*. *persicae* may accomplish continuous asexual reproduction under a stable climate and food supply [47]. *Acyrthosiphon pisum*, which is closely related to *M*. *persicae*, is known to induce asexual reproduction by thelytokous parthenogenesis at high temperatures [48, 49]. In general, because the numbers of prey and predators in complex environments exhibit continuous oscillation [50], it is possible that *M*. *persicae* continuously proliferated in the smart farm due to the blocking of access of their predators, the suitable artificial light conditions, and the maintenance of stable temperature inside the facility for their proliferation (average of 11°C).

The number of aphids under RB(1:1) was higher in December and March than in January and February (Fig 2B). In contrast, it was higher under RB(5:1) in January and February than in December and March (Fig 2B). Under RB(10:1), it was highest in February (Fig 2B). Changes in the number of aphids over time are generally associated with the temperature, aphid growth period, aphid sexual/asexual reproductive period, aphid life span, and alate forms of plant-to-plant migration [44, 47, 48, 51]. The different oscillating patterns of the number of aphids according to these light conditions imply that the number of aphids may also be affected by the light conditions.

The number of aphids and the Pn of the host plant *E*. *japonicum* showed opposite results depending on the light conditions (Figs 2 and 3). The number of aphids was highest under RB(1:1) and lowest under RB(10:1) (Fig 2B). Conversely, the Pn of *E*. *japonicum* was lowest under RB(1:1) and highest under RB(10:1) (Fig 3A). Therefore, with an increasing RB ratio, the Pn increased, whereas the number of aphids decreased. Additionally, although there were no differences in leaf densities between light conditions, the number of aphids attached per *E*. *japonicum* plant was the highest under RB(1:1) and lowest under RB(10:1), indicating that aphids prefer host plants with low Pn, regardless of the leaf toughness (Figs 3B and 4). This result may be attributed to the photosynthetic capacity of *E*. *japonicum* according to light quality and the complex responses to the predator-prey relationship between aphids and *E*. *japonicum*.

The Pn of plants can be affected by the ratio of red to blue light [38–40], and plants respond to herbivory through photosynthetic products [52]. Plant Pn is influenced by various physiological variables. Therefore, it is possible to manage environmental changes by actively controlling photosynthesis using various physiological variables within the limited photosynthetic capacity that can potentially occur when the environment changes [53]. However, because photosynthetic capacities can be altered by light stress [54], in environments where photosynthetic capacities are low, plants may find it difficult to cope with such situations by controlling their Pn when environmental pressures such as herbivory occur.

The light quality also directly can affect producing their secondary metabolites as well as Pn [55]. The blue light enhanced total phenolic contents of *Stevia rebaudiana* [56], and in *Brassica juncea*, the glucosinolate contents (GSLs) were directly affected on the white, red and blue single light [57]. These reports provide the insight the secondary metabolites, mainly using plant defense on their herbivores, of *E*. *japonicum* were also directly affected on the light quality.

The host plants belonging to the Brassicaceae family, which includes *E*. *japonicum*, respond to herbivory through primary and secondary metabolites [58]. Starch is one of the primary metabolites [52]. *Arabidopsis thaliana*, another member of the Brassicaceae family, transports sugar to tissues damaged by *M*. *persicae* aphids, resulting in a reduction in the number of aphids by interfering with the herbivory of *M*. *persicae* through osmotic effects [52].

Brassicaceae plants also produce secondary metabolites, such GSLs, for chemical protection [59–61]. *A*. *thaliana* accumulated GSLs in their leaves that were damaged by *M*. *persicae*, and when GSL production was high, the number of aphids decreased [62]. Therefore, the higher the Pn of *E*. *japonicum* when infected with aphids, the more primary and secondary metabolites may be produced, resulting in a more efficient defense system against aphids. In the present study, under the light conditions of RB(5:1) and RB(10:1) (compared to RB(1:1)), *E*. *japonicum* could more easily attain photosynthetic capacity and subsequently improve the Pn more easily to defend against herbivory by aphids. Therefore, there were fewer aphids sucking the sap of *E*. *japonicum* and fewer leaves attached to the aphids (Fig 3B).

Factor analysis revealed that the Pn of *E*. *japonicum* had a negative effect on the number of apterous aphids and the number of aphids on the upper and lower sides of *E*. *japonicum* leaves (Table 2); considering that the distribution ranges of the factor scores for each light condition were divided, there was a difference in these effects depending on the light quality (Fig 5).

Given that the proportion of alate aphids to apterous aphids observed during the study period was very small (5.34%) and the number of alate aphids was not related to Pn, regardless of the settled position of aphids on the *E*. *japonicum* leaf, Pn is considered to have a negative effect on apterous aphids rather than alate aphids. In this reason, the apterous form is thought to the main aphid form that causes herbivorous damage to *E*. *japonicum* rather than the alate form.

When the number of aphids attached to the lower side of the leaf increased, the number of alate aphids also increased (Table 2). However, because Pn was not related to the number of alate aphids, this result may be caused by the increase in aphid population density due to the production of apterous aphids on the lower side of the leaf [51].

## Conclusions

In summary, unlike general crop production facilities, smart farms are sealed from the external environment and are not affected by the weather, and the internal environment can be artificially controlled. Maintaining a constant temperature inside a smart farm during winter can sustain the growth of the perennial *E*. *japonicum*. Therefore, using *E*. *japonicum* as a host for aphids, even in winter, allows *M*. *persicae* to continuously produce progeny rather than wintering with eggs, as in the wild environment, thereby enabling the growth of aphids.

The number of aphids increased with lower Pn, regardless of the leaf density of the host plant *E*. *japonicum*. The Pn of *E*. *japonicum* was negatively affected by the number of apterous *M*. *persicae* aphids and the number of aphids on the upper and lower side leaves of *E*. *japonicum*. Moreover, the number of alate aphids was positively affected by the number of apterous aphids attached to the lower side of the leaves rather than Pn.

Therefore, it is possible to mass-produce aphids for augmentative biocontrol agents in the winter season by maintaining a constant temperature within a smart farm with limited access to predators and supplying suitable light quality that can lower the Pn of the perennial host plant, *E*. *japonicum*.

## Supporting information

S1 Table The number of aphids monthly observed by light conditions in a smart farm facility. (XLSX)

S2 Table The number of aphids monthly observed of each leaf of *Eutrema japonicum* by light condition in a smart farm facility. (XLSX)

S3 Table Averages and standard deviations of net photosynthetic rates of *Eutrema japonicum* in March by light conditions in a smart farm facility. (XLSX)

S4 Table Net photosynthetic rate (Pn) of *Eutrema japonicum* in March by light condition in a smart farm facility. (XLSX)

S5 Table The number, averages and standard deviations of leaves of *Eutrema japonicum* by light conditions in a smart farm facility. (XLSX)
